# Emerging Therapeutic Potential of Fisetin for Nephrotoxicity, Kidney Injury, and Nephropathy: A Systematic Review

**DOI:** 10.2174/0115733998369114250512094027

**Published:** 2025-06-03

**Authors:** Saeed Mohajeri, Alizamen Salehifard Jouneghani, Saeid Heidari-Soureshjani, Catherine MT Sherwin, Faramarz Beigi

**Affiliations:** 1 Department of Pediatrics, Shahrekord University of Medical Sciences, Shahrekord, Iran;; 2 Department of Pediatrics, School of Medicine, Shahrekord University of Medical Sciences, Shahrekord, Iran;; 3 Modeling in Health Research Center, Shahrekord University of Medical Sciences, Shahrekord, Iran;; 4 Department of Pediatrics, Pediatric Clinical Pharmacology and Toxicology, Wright State University Boonshoft School of Medicine, Dayton Children's Hospital, One Children's Plaza, Dayton, OH 45324, USA;; 5 Department of Urology, School of Medicine, Shahrekord University of Medical Sciences, Shahrekord, Iran

**Keywords:** Fisetin, nephroprotective, nephrotoxicity, kidney injury, diabetes, nephropathy

## Abstract

**Introduction/Objective:**

Kidney diseases cause high morbidity and mortality worldwide. This study investigated the mechanistic effects of Fisetin (FIS) on nephrotoxicity, kidney injury, and nephropathy induced by drugs, toxic chemicals, diabetes, lupus, diet, ureteral obstruction, and ischemic situations.

**Methods:**

To identify pertinent articles published before Oct 1, 2024, a comprehensive electronic search was performed across several databases, including MEDLINE/PubMed, Embase, Cochrane Library, Web of Science, and Scopus. After establishing clear inclusion and exclusion criteria, studies that met the research objectives were selected. Data were extracted and analyzed, documenting study characteristics, methodologies, and biological mechanisms.

**Results and Discussion:**

Antioxidant benefits were evident with increased levels of endogenous antioxidant enzymes and NQO1, alongside reduced oxidative stress markers such as 8-OHdG and MDA. Enhanced mitochondrial function, including improved respiration, ATP synthesis, and antioxidant capacity, further supported cellular resilience. Anti-inflammatory effects were marked by reduced pro-inflammatory cytokines, macrophage and neutrophil infiltration, and inhibited pathways like NF-κB and MAPK. Anti-apoptotic actions included decreased levels of pro-apoptotic proteins. FIS also reduced fibrotic markers and pathways such as TGF-β/SMAD, mitigating excessive ECM buildup. Additionally, modulation of metabolic pathways was observed, including decreased glucose and lipid profiles and improved insulin sensitivity. Kidney function and structural integrity were preserved with reduced levels of nephrotoxic agents.

**Conclusion:**

Preclinical studies revealed that FIS demonstrates promising protective effects against kidney toxicity, renal injury, diabetes, and lupus-induced nephropathy. However, more clinical studies are needed in this field to determine effective and safe doses.

## 1 INTRODUCTION

Kidney diseases have high mortality and morbidity around the world. Chronic kidney disease (CKD) affects more than 800 million individuals, especially those who experience diabetes mellitus and hypertension [1]. CKD and acute kidney diseases (AKD) may occur due to various diseases, including diabetes, cardiovascular diseases (CVD), inflammatory diseases, drugs, and toxins [2-4]. For instance, various medications prescribed for the management and treatment of various conditions, such as hypertension, cancer, and diabetes, can lead to glomerular filtration rate (GFR) alteration, tubular cell toxicity, interstitial nephritis, tubular obstruction or crystal nephropathy of the kidneys [5, 6]. Kidney inflammation, nephrotoxicity, ureteral obstruction, and renal ischemic reperfusion injury (IRI) significantly affect renal physiology and function by altering hemodynamics, GFR, and renal metabolism, inducing histological malformations, disturbing biochemical indices, and renal fibrosis [7-9]. Patients with CKD experienced lower quality of life in all aspects of health-related quality of life (HRQOL) compared to the control group [10]. CKD and kidney replacement therapy represent a significant global economic burden, with the financial impact intensifying proportionately with the progression of the disease's severity [11, 12]. Moreover, studies indicate that certain drugs and various kidney disorders can lead to complications in essential organs, including the heart and gastrointestinal system, in addition to affecting the kidneys directly [13-16]. Therefore, it prevents disease progression and alleviates many of these complications. Studies indicated that one of the preventive strategies for acute and chronic kidney disorders is to use the antioxidant, anti-inflammatory, and anti-apoptotic capacity of phytochemicals [17, 18]. 3,3′,4′,7-tetrahydroxy flavone or fisetin (FIS) classified as flavonoid is naturally found in various fruits and vegetables, including apples, mangoes, persimmon, grapes, kiwis, cucumber, onion, and tomatoes [19, 20]. FIS revealed nephroprotective potential in previous studies due to its antioxidant and anti-inflammatory properties. It also revealed modulatory effects on blood urea nitrogen (BUN) and creatinine (Cr) [21-23]. Recent evidence shows that the use of FIS has been proposed as a new and effective treatment strategy against the progression of renal fibrosis [24]. Considering the rising incidence of nephrotoxicity caused by drugs and toxic agents [1, 25], together with the growing prevalence of cancer, diabetes, and hypertension—key contributors to nephropathy and kidney fibrosis [26-28]—it is crucial to investigate these issues further. To date, however, no comprehensive review has examined the effects of fisetin (FIS) on nephrotoxicity, kidney injury, and disease-induced nephropathy in diabetes and lupus. Therefore, this systematic review was designed to fill that gap in our understanding.

## 2 MATERIALS AND METHODS

### 2.1 Search Strategy

On Oct 1, 2024, a thorough electronic search was conducted across multiple reputable databases, including PubMed/MEDLINE, Scopus, Web of Science, Embase, and the Cochrane Library. This meticulous search aimed to identify relevant studies using a combination of Medical Subject Headings (MeSH) terms and widely recognized keywords derived from previous research. The search strategy was conducted based on precisely selected keywords including: (“Fisetin” OR “3,7,3′,4′-tetrahydroxyflavone”) AND (“acute kidney injury” OR “chronic kidney disease” OR “acute renal injury” OR “kidney injury” OR “renal injury” OR “nephrotoxicity” OR “renal toxicity” OR “renal failure” OR “kidney dysfunction” OR “kidney failure” OR “nephropathy” OR “renal fibrosis” OR “drug-induced nephrotoxicity” OR “nephrotoxic agents” OR “chemotherapy-induced nephrotoxicity” OR “nephrotoxic drugs” OR “renal inflammation” OR “renal hypoxia” OR “renal ischemia” OR “nephropathy”).

In this systematic review, we conducted a comprehensive search to identify relevant publications, carefully examining studies and previous review articles. As we searched the publications, we refined our search strategy to ensure comprehensive coverage of all pertinent publications related to our systematic review. To preserve the integrity of our dataset and screening, we imported the selected peer-reviewed articles into EndNote 21.0.1, dated Jul 25, 2023, from Thomson Reuters. Through this meticulous process, we systematically identified and removed duplicate entries, ensuring that our final selection accurately represented the existing research full text.

### 2.2 Selection Criteria

We have included all the clinical and experimental studies investigating FIS's effects on nephrotoxicity, kidney inflammation, and fibrosis induced by drugs, toxic chemicals, disorders, diet, ureteral obstruction, and ischemic situations. Our selection criteria included clinical trials and preclinical (*in vivo* and *in vitro*) studies that meet the PICO criteria, with the population being patients/animals/cells with induced nephrotoxicity, kidney injury, and nephropathy (due to diabetes and lupus), the current treatment for the mentioned disease, and the administration of FIS considered as intervention, with patients/animals and cells without kidney disease as control. Furthermore, we considered the reported mechanism of FIS on kidney disorders as outcomes. We have excluded various types of studies, including meta-analyses, systematic reviews, editorials, and case reports, as well as studies lacking original data, those published only in abstract form, conference poster presentations, unpublished study protocols, articles in non-English languages, and letters to the editors. During the screening phase, selected articles underwent a comprehensive full-text evaluation to determine their eligibility, with clear justifications documented for each exclusion. The publications were independently assessed for their quality.

### 2.3 Screening Process

Two reviewers evaluated the titles and abstracts of the studies according to predefined inclusion and exclusion criteria. Potential studies that met the inclusion criteria were identified, and their full-text articles were retrieved. The same investigators then independently assessed the full text of the articles to determine their eligibility based on the predefined criteria. To resolve any disagreements during the evaluation process, we engaged a third reviewer to assist in reaching a consensus.

### 2.4 Data Extraction

A standardized form to collect the required data, including author (s), publication year, type of the study, chemical/disorders-induced nephrotoxicity and nephropathy, studied population /animals/cells, treatment details (dosage, duration) and main suggested mechanisms, and safety were extracted.

### 2.5 Narrative Synthesis

The data synthesis process involved creating a narrative summary of the results from the studies included in the analysis. The main focus was investigating the effect of FIS on nephrotoxicity, kidney injury, and nephropathy and exploring the mechanisms underlying its action.

### 2.6 Reporting Guidelines

We adhered to the Preferred Reporting Items for Systematic Reviews and Meta-Analyses (PRISMA) 2020 guidelines to guarantee that our reporting of the systematic review process is thorough, accurate, and transparent. This approach allows us to provide a detailed account of our methodology, findings, and overall rigor in reviewing relevant literature.

## 3 RESULTS

### 3.1 Search Results

The search strategy is outlined in Fig. (**1**) using a PRISMA flowchart. Initially, 277 titles and abstracts were identified through electronic searches from February 2003 to August 2024. Of these, 209 articles were removed due to duplicate entries. An additional seven were excluded for various reasons: one was an editorial [24], one was not in English [29], in a few, the full text was not available (poster papers) [30-33], and one did not include our indices [34]. After screening the articles, 27 publications were included in this study based on our inclusion and exclusion criteria [35-61].

### 3.2 Description of the Included Studies

All included studies were preclinical studies that classified as drugs and chemical-induced nephrotoxicity, renal IRI, diabetes, lupus, and other disorders-induced nephropathy (Tables **1**-**3**). The compound demonstrates significant nephroprotective properties through various mechanisms. It mitigates oxidative stress by reducing DNA damage markers such as 8-OHdG, enhancing antioxidant enzymes, and improving mitochondrial function and adenosine triphosphate (ATP) synthesis. Its anti-inflammatory properties are evident in the reduction of macrophage infiltration, inhibition of NF-κB, and suppression of pro-inflammatory cytokines. The compound also exhibits anti-apoptotic effects by decreasing apoptosis-related protein levels. At the same time, its anti-fibrotic actions and anti-ferroptotic properties. Histopathological improvements include lower kidney injury markers and structural regeneration. Additionally, the compound modulates kidney biochemical markers, enhancing electrolyte and micronutrient balance and lipid profiles. Metabolically, it supports glucose and lipid homeostasis by elevating vital glycolytic enzymes and reducing blood glucose levels and HbA1c. Furthermore, it enhances kidney structure by upregulating nephrin and podocin and promotes nephroprotection *via* microbiota-derived tryptophan metabolites like L-kynurenine.

### 3.3 Natural Product's Role in Pharmacology

For many years, natural products and their derivatives, such as phytochemicals, have demonstrated their potential as effective drug candidates for various disorders [62]. Natural products potentially provide novel compounds that can be transformed into medications due to the variety of their chemical structures [63]. Progress in engineering strategies, analytical tools, and genome mining has turned drug discovery into a multifaceted process that includes computational design and testing, followed by laboratory evaluation of promising drug candidates [63, 64]. The main drugs derived from natural products that have received approval for clinical application are anti-infective medications, cancer treatment agents, CVDs, and pain relief medications (Table **4**).

### 3.4 FIS Chemical Structure

FIS is a plant metabolite compound found in many fruits and vegetables, known as 3,3′,4′,7-tetrahydroxyflavone. FIS's molecular formula is C_15_H_10_O_6_ and a molecular weight of 286.24 g/mol. It is often present in various fruits and vegetables, including strawberries, grapes, onions, cucumbers, and persimmons [72] (Fig. **2**).

Fis has various pharmacological properties, including its normal cells cytoprotection, antioxidant, anti-inflammatory, chemotherapeutic, chemopreventive, antiangiogenic, and neuroprotective effects [73, 74]. It is orally bioavailable and by upregulating glutathione (GSH) levels and inhibiting pro-inflammatory mediators, including tumor necrosis factor-alpha (TNF-α), interleukin-1 beta (IL-1β), IL-6, nuclear factor kappa B (NF-κB) indicate antioxidant and anti-inflammatory properties [73, 75].

### 3.5 FIS’s Nephroprotective Properties

#### 3.5.1 Antioxidant Properties

The renal system is particularly vulnerable to oxidative stress, with reactive oxygen species (ROS) as a critical factor in disease and dysfunction. Research indicates a significant imbalance between prooxidant and antioxidant activities in renal diseases, which often progress undetected due to the absence of early symptoms [76]. Molecular changes, such as increased ROS production from the mitochondrial respiratory chain and nicotinamide adenine dinucleotide phosphate (NADPH) oxidases, occur before clinical symptoms and can worsen renal damage [77]. Studies that investigated the effects of FIS on nephrotoxic mice and diarrheal mice revealed that it can improve NAD(P)H oxidoreductase 1 (NQO1), NADH and reduce NADH oxidase in kidney injuries [35, 58]. Given the kidneys' high mitochondrial content and substantial blood flow, oxidative stress proves especially detrimental, exacerbating renal function decline and disease progression [76].

FIS enhances intracellular GSH, superoxide dismutase (SOD), and catalase (CAT) levels, protecting against ROS activity [20]. In contrast, malondialdehyde (MDA) is a lipid peroxidation marker, signifying oxidative harm to cell membranes. Increased MDA levels suggest heightened oxidative stress and kidney damage in cases of nephrotoxicity [78]. Various studies have shown that FIS can improve glutathione Reductase (GR), Glutathione S-Transferase (GST), GSH, GSH/GSS, CAT, SOD, glutathione peroxidase (GPx), capacities in mice with kidney injuries [35, 40, 44, 58, 61].

In primary rat neurons, FIS counteracts oxidative stress caused by the peroxynitrite donor SIN-1, stabilizing ERK1/2 and c-myc phosphorylation, nuclear factor erythroid 2-related factor 2 (Nrf_2_) levels, glutamate cysteine ligase, GSH concentration, and cell viability. Additionally, FIS promotes heme oxygenase-1 (HO-1) expression *via* Nrf_2_ activation, which reduces hydrogen peroxide-induced cell apoptosis, a protective effect diminished by HO-1 inhibition [20, 79]. These mechanisms help mitigate oxidative damage to renal tissues caused by nephrotoxic drugs, such as cisplatin and gentamicin, and environmental toxins like lead, cadmium, and arsenic. FIS activates the Nrf_2_ pathway, which regulates antioxidant response genes [76]. An *in vivo* and *in vitro* study by Berköz *et al.*, using male Wistar albino rats with lead acetate-induced nephrotoxicity, revealed that FIS enhanced the expression of Nrf_2_ and HO-1 [40]. Qian *et al.* indicated that 20 mg/kg bw/day of FIS for 8 weeks also increased Nrf_2_/ HO-1/GPX4 expressions [59]. The activation of Nrf_2_ is instrumental in reducing oxidative stress, a significant contributor to nephrotoxicity induced by drugs and toxins.

Moreover, indicators of lipid peroxidation, such as Receptor for Advanced Glycation End-products (RAGE), 4-hydroxynonenal (4-HNE), and conjugated dienes, are effectively reduced by FIS in cisplatin-induced nephrotoxic, ureteral obstruction, and diarrheal mice [44, 57, 58].

Furthermore, the kidney's susceptibility to DNA-damaging chemotherapeutic agents and its role in the incidence of acute kidney injury, kidney ciliopathies, and DNA damage is well recognized. Toxic agents and renal IRI in humans can also indicate genotoxic stress, impair DNA repair, and have detrimental DNA crosslinks, breaks, and transcription in kidney tissue [80]. ROS-caused DNA damage due to drugs, renal IRI, and sepsis are the most prevalent forms of DNA damage linked to the development of AKI and CKD. When DNA lesions occur, cells may undergo repair or be eliminated through cell death if repair efforts are unsuccessful. DNA damage typically activates or increases the production of DNA repair proteins. However, if the damage is too extensive to be fixed, the organism can initiate apoptosis to prevent heavily damaged cells from acquiring mutations that could lead to cancer [81]. Administration of 20 mg/kg b.wt, ip FIS for 30 minutes before the surgery in male Wistar rats with renal IRI can reduce DNA fragmentation [56]. Koriem *et al.* demonstrated that FIS at 50 mg/kg for 4 weeks potentially reduced kidney DNA damage, which is vital for sustaining electrolyte balance, cellular function, and overall kidney health [58]. About 25 mg/kg/ day of FIS 1 hour before surgery in ureteral obstruction mice for 7 days reduced 8-OHdG as a biomarker commonly used to assess oxidative DNA damage [57].

#### 3.5.2 Improve Mitochondrial Function

The kidney is a mitochondria-rich organ with high energy demands [82]. Mitochondrial injury frequently occurs in drug-induced toxicity cases. The kidney damage associated with certain medications may be due to the elevated mitochondrial density in the cytoplasm of tubular cells and the diverse range of drug transporters significantly expressed on both the basolateral and brush border membranes of proximal tubular cells [83]. The disruption of mitochondrial function through mitophagy disruption, mitochondrial dynamics, and mitochondrial fusion are linked to kidney injuries. When mitochondrial function is impaired, it disturbs the cellular redox balance, which can eventually lead to cell apoptosis. Additionally, mitochondrial DNA released from damaged cells can serve as damage-associated molecular patterns, attracting immune cells and intensifying the inflammatory response, thereby aggravating kidney injury [84]. Two preclinical studies revealed that FIS significantly improved mitochondrial antioxidant capacity and mitochondrial morphology in ureteral obstruction and cisplatin-induced nephrotoxic mice [35, 61].

Polyphenols play a significant role in alleviating mitochondrial dysfunction and enhancing mitochondrial functionality. They achieve this by reducing mitochondrial ROS, promoting mitochondrial dynamic and biogenesis and ATP production, improving mitochondrial structure, and improving mitophagy. A diverse array of natural compounds has been found to influence mitochondrial function, including activators of AMP-activated protein kinase (AMPK), Sirtuins, Nrf_2_, and various endogenous antioxidants [85]. Prem *et al.* conducted on male Wistar rats with renal IRI, 20 mg/kg b.wt, ip of FIS for 30 minutes before the surgery, improved mitochondrial and ATP synthase functions [56]. A high dose of 1.25 mg/kg of FIS improves mitochondrial antioxidant capacity and mitochondrial respiration, including NADH dehydrogenases, succinate dehydrogenase (SDH), and cytochrome c oxidase in mice administered cisplatin-induced nephrotoxicity [35].

#### 3.5.3 Anti-inflammatory Effects

The immune system and inflammatory cytokines play a critical role in renal pathological progression, including morphological or functional alteration in the epithelium and endothelial cells, disruption repair and remodeling mechanisms, electrolyte imbalances, disrupt kidney hemodynamic, glomerular, and renal tubular injury [86, 87]. These conditions are aggravated by underlying diseases such as diabetes and hypertension [88]. Immune cells release interferon-gamma (IFN-γ), and TNF-α can either exacerbate or limit renal damage and renal fibrosis due to inflammation and oxidative stress. Typically, cytokines contribute to renal disease by enhancing the expression of endothelial adhesion molecules and chemokines, increasing immune cell infiltration, and activating NF-κB, a transcription factor related to a release of TNF-α, IL-1β, IL-6, IL-8, and IL-12, which are elevated in nephrotoxic conditions [86].

Hada *et al.* indicated that FIS (100 μM of FIS for 3 h) reduces macrophage infiltration by downregulating key markers such as uPA, uPAR, matrix metalloproteinase-2 (MMP-2), and MMP-9, which are integral to tissue remodeling and the migration of immune cells of BALB/c mice. This attenuation of macrophage presence minimizes renal tissue damage while concurrently reducing neutrophil infiltration, which mitigates oxidative and enzymatic injury factors critical in preventing acute inflammatory responses [38].

As various experimental studies have shown, the central mechanism by which FIS exerts its protective effects is the inhibition of NF-κB, a crucial regulator of inflammation. This inhibition results in decreased levels of pro-inflammatory cytokines, including TNF-α, IL-1β, IL-6, IL-10, and IL-18 [35, 37, 38, 40, 47-49, 51, 58, 60]. Furthermore, Ren *et al.* reported that antagonizes toll-like receptor 4 (TLR4), which further inhibits NF-κB and mitogen-activated protein kinase (MAPK) signaling pathways, including p38, ERK1/2, and JNK, effectively inhibiting downstream activation of inflammatory mediators by administration 100 mg/kg of FIS orally applied for 3 consecutive days in lipopolysaccharide-induced nephrotoxicity male C57BL/6 J mice [37].

Furthermore, mitochondrial reactive oxygen species (mtROS) also trigger inflammatory mechanisms by acting as ligands for receptors such as the NOD-like receptor protein 3 (NLRP3) inflammasome and toll-like receptors (TLRs). The mtROS constitute a significant damage-associated molecular pattern (DAMP) that induces renal inflammation, although other mechanisms may also contribute to the inflammatory response [76]. Certain drugs and toxins can activate immune and parenchymal cells, reduce renal macrophage infiltration, activate the NLRP3 inflammasome, and cause irreversible renal tissue damage and functional loss [8]. Additionally, FIS has been demonstrated to reduce NLRP3 inflammasome activation, which diminishes the release of pro-inflammatory cytokines, thereby safeguarding kidney cells from exacerbated inflammatory injury [37]. In addition to these mechanisms, FIS reduces the levels of chemokines such as CXCL-1, CXCL-2, and CCL3, which attract immune cells to inflamed tissues, thereby preventing excessive immune cell accumulation within the mice kidneys with lupus erythematosus [47]. The downregulation of vascular cell adhesion molecule-1 (VCAM-1) by FIS (30 mg/kg b.wt) further limits the adhesion of immune cells to the endothelium, restricting their migration into renal tissues in LDLR^−/−^ mice with diabetes [60]. Moreover, various studies revealed that by attenuating the effects of MCP-1 and MIP-2, FIS significantly decreases the recruitment of monocytes and neutrophils, effectively curtailing additional inflammatory cell infiltration [38, 47, 53]. FIS additionally targets specific inflammatory enzymes and proteins, such as cyclooxygenase-2 (COX-2) and its product, prostaglandin E2 (PGE2), implicated in inflammation and pain. By inhibiting COX-2 and PGE2, FIS diminishes the presence of pro-inflammatory mediators that may exacerbate renal inflammation [37, 38]. Furthermore, FIS inhibits inducible nitric oxide synthase (iNOS) and myeloperoxidase (MPO), reducing the production of excessive nitric oxide and reactive ROS, thus minimizing oxidative stress and tissue damage in renal cells due to chemical and drug toxicity. FIS also suppresses high mobility group box 1 (HMGB1), a critical inflammatory mediator in renal injury, diminishing its role in activating immune cells [35-38].

*in vivo* and *in vitro* studies demonstrated that FIS inhibits the activity of T-helper 17 (Th17) cells, consequently reducing levels of the pro-inflammatory cytokine IL-17, thereby contributing to a less aggressive inflammatory response. Additionally, FIS decreases IFN-γ levels, a cytokine known to exacerbate inflammation, particularly in autoimmune-related renal injuries, thereby preventing excessive inflammatory damage [47].

By antagonizing TGF-β1, FIS effectively prevents fibrosis, a critical consideration in chronic kidney disease, wherein inflammation frequently leads to scarring and functional decline. Furthermore, FIS’s (20 μM for 30 min) inhibition of ATP-competitive MKK4 contributes to the prevention of downstream inflammatory pathway activation, offering additional protection against renal injury in RAW264 cells treated with lipopolysaccharide [41].

#### 3.5.4 Anti-apoptotic Effect

Apoptosis is a vital mechanism in nephrotoxicity and kidney injury, primarily initiated by ischemic situations, drugs, heavy metals, and toxic chemicals. Regulated necrosis involves multiple specific pathways, such as pyroptosis, necroptosis, ferroptosis, and mitochondrial permeability transition-dependent necrosis. Each pathway is significantly affected by inflammation, tissue repair, and kidney disease progression [89]. These toxins cause stress in kidney cells, especially in renal tubular cells (mainly proximal tubule epithelial cells), and they activate mitochondrial and endoplasmic reticulum (ER) stress pathways. This activation leads to apoptosis by engaging caspases and subsequent inflammatory responses. The resulting cell apoptosis decreases kidney function by depleting functional cells and disrupting nephron structure, which can lead to AKI and, if unaddressed, progress to CKD [90, 91]. In addition to reducing tubule cell apoptosis through antioxidant and anti-inflammatory activities, FIS (25 mg/kg for 6 days in mice) also prevents tubular apoptosis and reduces injury and nephropathy of the kidney under stress [57].

DNA injury can activate B-cells and inhibit anti-apoptotic B-cell lymphoma family-2 (BCL-2) proteins. This inhibition facilitates the oligomerization and activation of BCL-2-associated X protein (BAX) and BCL-2 antagonist killer 1 (BAK), forming pores in the outer mitochondrial membrane. The permeabilization of the mitochondrial outer membrane (MOMP) results in the release of cytochrome c and the second mitochondrial activator of caspase (SMAC) [92].

Cytochrome c binds to apoptotic protease-activating factor 1 (APAF1) and pro-caspase-9, forming the apoptosome. Within this structure, procaspase-9 dimerizes and triggers a caspase cascade. The active caspase-9 heterodimer cleaves and activates the effector caspases -3 and -7, inducing apoptosis [92]. Sahu *et al.* administered 2.5 mg/kg of FIS, which was applied intraperitoneally for 7 consecutive days on male Sprague-Dawley rats with cisplatin-induced nephrotoxicity reduced cytochrome c protein, Bax, cleaved caspase-3, cleaved caspase-9, and p53 expressions [35]. Moreover, Ren *et al.* administered 100 mg/kg of FIS orally for 3 consecutive days on male C57BL/6 J mice with nephrotoxicity. They found the flavonoid compounds Bcl-2, Bax, and caspase-3 [37]. So, targeting apoptotic pathways, such as employing caspase inhibitors and antioxidants, shows promise for mitigating kidney damage.

#### 3.5.5 Anti-fibrotic Activity

Kidney fibrosis is a complex interplay of renal structural and functional changes characterized by several defining features, including tubular atrophy, chronic inflammatory processes within the interstitium, fibrogenesis, glomerulosclerosis, and a notable decrease in vascular density [93].

Renal inflammation is crucial in triggering fibrosis, evident in acute and chronic kidney injury, where cytokine release and inflammatory cell infiltration drive the epithelial to mesenchymal transition (EMT), ultimately resulting in renal fibrosis and dysfunction [94]. Chronic inflammation, a defining characteristic of CKD, often initiates fibrosis. This inflammatory response involves epithelial and endothelial cells that produce mediators such as cytokines and chemokines, which recruit various inflammatory cells, including lymphocytes, neutrophils, eosinophils, basophils, mast cells, and macrophages [4, 95]. These immune cells, fibroblasts, endothelial cells, and Fibrocytes release transforming Growth Factor Beta 1 (TGF-β1). This potent fibrogenic factor activates fibroblasts and boosts the synthesis of Extracellular Matrix (ECM) proteins [96]; in patients with AKI and CKD, elevated levels of soluble amphiregulin in urine correlate positively with fibrosis markers in kidney biopsies. Myofibroblasts significantly contribute to ECM protein production in the context of cardiac fibrosis. At the same time, immune and renal cells (epithelial and tubule cells) also release cytokines, including TNF-α, IL-1β, IL-10, IL-22, TGF-β1, and colony-stimulating factor-1 (CSF-1), which promote renal fibrosis [94, 96].

Moreover, angiotensin II (Ang-II) is the principal active structure of the renin-angiotensin system (RAS), recognized for its role in promoting collagen type 1 (COL1) and fibronectin synthesis. It stimulates Smad signaling pathways through a TGF-β-independent mechanism. Furthermore, Ang-II activates TGF-β1 pathways, underscoring the crucial role of the RAS in kidney fibrosis [97]. By suppressing M2 macrophages, FIS (25 mg/kg for six days in mice) reduces the release of pro-fibrotic cytokines and factors contributing to fibrosis. Furthermore, FIS interferes with the TGF-β1 signaling pathway, an essential mediator in fibrosis, blocking the phosphorylation of SMAD2 and SMAD3 proteins [57]. Another study reported that TGF-β1 typically activates these proteins to regulate the gene expression associated with ECM production, including collagen type I (COL1), fibronectin, and α-smooth muscle actin (α-SMA). By inhibiting this pathway in hyperuricemic nephropathy C57BL/6J mice, FIS at 100 mg/kg dosage for 4 weeks effectively decreases the synthesis of these ECM proteins, thereby preventing excessive ECM deposition, characteristic of fibrotic diseases [52].

#### 3.5.6 Anti-ferroptosis Effects

Ferroptosis is a recently recognized type of apoptosis, related to several diseases and leads to iron overload and nephrotoxicity [98, 99]. It affects metabolic and signaling pathways and has been associated with kidney disorders like CKD, AKI, ischemic organ damage, diabetic nephropathy, lupus nephritis, renal fibrosis, and clear cell renal cell carcinoma [100]. Ferroptosis is characterized by the accumulation of ROS and lipid hydroperoxides resulting from the failure of GPX4, mitochondrial iron metabolism, tetrahydrobiopterin (BH4), suppressor protein 1 (FSP1), and are essential antioxidant enzyme for cell survival [101]. This oxidative imbalance poses a significant threat to the kidneys, as renal tubular cells are particularly vulnerable to ROS and lipid peroxidation [102]. Key ferroptosis molecules, GPX4 and Nrf_2_, inhibit NLRP3 activation while receptor-interacting protein kinase 3 (RIPK3) and caspase-8 activation promote it, influencing necroptosis and pyroptosis. Additionally, p53 links apoptosis, autophagy, and ferroptosis by inhibiting SLC7A11 and promoting ferroptosis [101]. A study by Wang *et al.* indicated that FIS at 100 mg·kg^−1^·d^−1^, ig dosage inhibited mRNA and protein expression of ACSL4 (as key ferroptosis enzyme), COX2, and HMGB1, and improved GPX4 in CKD mice as well as in injured TCMK-1 cells.

Ferroptosis is closely associated with renal fibrosis in CKD, which accelerates tissue scarring and functional deterioration within the kidneys. TGF-β1, a potent pro-fibrotic cytokine that enhances oxidative stress, inflammation, and EMC deposition, deleteriously affects kidney function and fibrosis [100].

#### 3.5.7 Histopathological Changes

Nephrotoxicity and kidney injury lead to distinct histopathological alterations, primarily affecting the tubules, glomeruli, interstitium, and blood vessels. Tubular injury often manifests as necrosis, loss of brush borders, cellular sloughing, and dilation, possibly due to toxins or ischemic situations [103]. Pathological changes such as hypertrophy, mesangial expansion, and glomerulosclerosis are particularly evident in chronic or immune-mediated injuries in the glomeruli. The interstitial space commonly displays inflammation, fibrosis, and edema, while vascular changes can include endothelial damage, vasoconstriction, and arteriolar thickening [104]. Specific agents, including cisplatin, aminoglycosides, and heavy metals, are associated with characteristic damage patterns like proximal tubular necrosis or interstitial nephritis. Chronic injuries typically result in irreversible fibrosis and glomerulosclerosis, while early changes may be reversible with appropriate intervention [105]. FIS exhibits promising anti-fibrotic effects in the kidneys, particularly in mouse models of hyperuricemic nephropathy. It lowers the levels of neutrophil gelatinase-associated lipocalin (NGAL) and kidney injury molecule-1 (KIM-1), both recognized as biomarkers of renal damage and fibrosis [52]. According to Li *et al.* study, FIS (100 mg/kg for 4 or 8 weeks) reduces the expression of vimentin (VIM) in renal tubules, linked to fibrosis in C57BL/6 mice with cisplatin nephrotoxicity [39]. These actions enhance tubular regeneration and limit kidney hypertrophy, both essential for reversing fibrotic damage [44]. Moreover, FIS helps preserve kidney tissue architecture by diminishing the accumulation of toxic agents within the tissues, thereby offering protection against hyperuricemic-induced histological changes [53]. This preservation benefits the restoration of glomerular membrane integrity, reduces glomerular vacuolization, and maintains the typical structure of proximal and distal convoluted tubules [59].

#### 3.5.8 Enhancement of Autophagy

Autophagy is a well-conserved cellular process that targets intracellular components to lysosomes for breakdown. This process is crucial for sustaining kidney function and is linked to the development of renal diseases [106]. Renal IRI, toxic chemicals, immunological response, and oxidative stresses can trigger the process of autophagy in renal epithelial cells, altering the progression of several kidney disorders [107]. In pathological circumstances, autophagy helps in cellular adjustment; however, triggering autophagy following renal injury might not be adequate protection, particularly in IRI situations and nephrotoxicity [108]. Autophagy is a conserved process that degrades intracellular materials *via* lysosomes and is crucial for kidney function. During AKI, autophagy is activated in renal tubular cells to remove damaged organelles and protein aggregates, helping maintain cell viability and function. While initially protective, prolonged autophagy after AKI may impair kidney recovery, leading to tubular degeneration and a pro-fibrotic state that contributes to declining renal function [106]. FIS was applied to a streptozotocin-induced diabetic nephropathy mouse model, improving the mRNA expression levels of the CDKN1B/P70S6K pathway in high-glucose cultured podocytes that were treated with 30 µM of FIS for 48 hours [54].

#### 3.5.9 Modulate Kidney Biochemical Markers

Nephrotoxins and certain medications can cause kidney damage, with BUN and serum Cr levels commonly used to evaluate renal toxicity. However, recently identified biomarkers provide greater sensitivity and specificity, allowing for a more precise localization of kidney injury. In the bloodstream, markers such as KIM-1, cystatin C, NGAL, and OCT2 are more effective than BUN and Cr in detecting nephrotoxin-induced AKI [109, 110]. Ren *et al.*, in their study, reported that FIS at 100 mg/kg dosage for 4 weeks effectively reduced urea, BUN, and Cr and improved renal function [52]. Furthermore, another study reported that 200 mg/kg of FIS reduced urea, BUN, and Cr in lead-induced nephrotoxicity in mice [40]. Pharmacological agents that target these pathways, such as antioxidants, can help restore hemodynamic balance, enhance renal blood flow, and mitigate injury progression by protecting vascular function and maintaining GFR at a normal level [111]. In the study by Maher *et al.*, FIS administered at 40 mg/kg dosage for 6 weeks in C57BL/6-Ins2 (Akita) can reduce albuminuria [44].

#### 3.5.10 Regulating Gut Microbiota

The microbiome significantly contributes to CKD, AKD, degradation of oxalate, and kidney transplants by impacting the functionality of the intestinal barrier, moderating both local and systemic inflammation (diabetes, lupus, *etc.*), regulating the generation of metabolic substances, and influencing immune responses [112]. An *in vivo* study by Ren *et al.* revealed that administration of 50 and 100 mg/kg of FIS for 4 weeks on C57BL/6J mice could modulate the structure and composition of gut microbiota and improve kidney health through its interaction with tryptophan metabolites produced by gut microbiota, particularly L-kynurenine. These metabolites are linked to FIS's protective effects on the kidneys and are mediated by the aryl hydrocarbon receptor (AHR), which binds L-kynurenine in the kidney. In hyperuricemic mice, AHR expression was elevated; however, this expression decreased in FIS-treated mice, highlighting FIS's role in modulating the gut-kidney interaction and providing a comprehensive protective effect against nephropathy [52].

In general, (Fig. **3**) demonstrates the mechanisms of Fis's effect on nephrotoxicity caused by drugs and chemicals.

#### 3.5.11 Attenuate Nephrotic Syndrome

Nephrotic syndrome is a clinical condition marked by a significant protein loss in the urine, resulting in low blood Alb levels. This leads to elevated peripheral edema, hyperlipidemia, hypoalbuminemia, proteinuria, glomerulosclerosis, imbalance of body fluid, thrombosis, membraneous nephropathy, and chronic renal dysfunction [113]. The condition is caused by increased permeability of the damaged basement membrane in the kidney's glomerulus. This permeability issue can stem from either a primary kidney-specific disease or secondary factors, including congenital infections, diabetes, systemic lupus erythematosus, neoplasia, or specific medications [44].

Qian *et al.* reported that the antioxidant properties of FIS (5, 10, 20 mg/kg) are notably highlighted by its activation of the Nrf_2_/HO-1/GPX4 pathway, which plays a crucial role in cellular defense against oxidative damage in mice after 8 weeks. Activating Nrf_2_ enhances the expression of HO-1 and GPX4, essential enzymes that safeguard kidney cells, particularly podocytes, from ferroptosis, a form of cell necrosis driven by lipid peroxidation. This protective effect on podocytes is vital for maintaining the structure and function of the kidney's filtration system [59]. In addition to activating the Nrf_2_ pathway, studies revealed that FIS increases the levels of several other antioxidant molecules, including GSH/GSSG ratio, GPx, CAT, SOD, and NADPH. So, FIS neutralizes free radicals and maintains redox balance. Moreover, the compound lowers MDA, a marker of lipid peroxidation that signifies cell membrane damage from oxidative stress. By reducing MDA levels, FIS helps to prevent damage to kidney cell membranes, thereby preserving cell integrity and function [58, 61].

FIS reduces levels of RAGE (Receptor for Advanced Glycation End-products) and serum amyloid A, which are involved in inflammatory and immune responses related to renal disease. FIS inhibits pro-inflammatory cytokines such as TNF-α, IL-1β, IL-6, IL-10, and IL-18, as well as the NLRP3 inflammasome, all of which contribute to chronic kidney inflammation and damage [44, 48, 51, 54, 58]. Moreover, 100 mg/kg of FIS for 30 weeks in mice potentially inhibited interferon-gamma (IFN-γ) and reduced excessive immune activation, which can otherwise exacerbate kidney inflammation and damage [47]. Additionally, 30 mg/kg of FIS reduces the expression of VCAM-1 and MCP-1, which promote immune cell infiltration in damaged kidney regions, further protecting against immune-driven injury [60]. Zhang *et al.* in their study revealed that 10 mg/kg of FIS also modulates critical inflammatory pathways, including iRhom2/NF-κB and TAK1/p38MAPK/MK2, linked to cellular stress and inflammation in kidney diseases [49].

This flavonoid compound in 30 mg/kg b.wt dosage for 6 and 12 weeks also attenuates fibrosis by inhibiting the expression of CD36, a protein involved in lipid accumulation and inflammation within kidney cells. FIS's mechanism of action prevents the generation of ECM by targeting key pro- fibrotic factors such as vascular endothelial growth factor A (VEGFA), fibronectin, and collagen, which contribute to kidney scarring and tissue rigidity [60].

Ju *et al.* also revealed that FIS acts on pro-fibrotic M2 macrophages that promote fibrotic tissue alterations, diminishing their impact on kidney damage in obstructive nephropathy mice. By inhibiting the phosphorylation of SMAD2 and SMAD3 triggered by TGF-β1, a significant contributor to fibrosis, FIS disrupts critical signaling pathways associated with renal fibrosis [57]. It also preserves against tubular ferroptosis, a form of cell necrosis linked to lipid peroxidation, by reducing ACSL4 expression, thereby safeguarding kidney tubules from damage [61]. Furthermore, FIS diminishes inflammatory responses and fibrogenesis caused by adenine or TGF-β1, limiting chronic inflammation and injury in kidney tissue [61].

Furthermore, FIS has demonstrated the ability to reduce protein loss in urine, a key indicator of enhanced kidney function, while increasing serum Alb levels. This favorable balance in protein levels and reduced markers such as serum urea and Cr further indicate improved renal function and decreased kidney stress [51]. Moreover, Kumari *et al.* reported that FIS can alleviate complications associated with diabetes-related nephropathy and atherosclerosis is a significant finding. Its capacity to lower urine glucose levels and improve key indicators such as serum urea, uric acid, and Cr not only aids in managing diabetic kidney damage but also mitigates the exacerbating impact of atherosclerosis on nephropathy [60].

Additionally, electrolyte imbalance is another biomarker for detecting kidney injury [110, 114]. FIS 's role in regulating vital electrolytes, including sodium, potassium, and chloride, which are critical for maintaining kidney filtration and blood pressure equilibrium, further underscores its versatility. Koriem *et al.* demonstrated that FIS (50 mg/kg of FIS for 4 weeks) has been shown to enhance Na^+^/K^+^-ATPase activity, which is vital for sustaining electrolyte balance, cellular function, and overall kidney health [58]. FIS significantly impacts lipid profiles in metabolic health. It reduces harmful lipids such as low-density lipoprotein and VLDL while increasing HDL levels. This contributes to a balanced lipid profile and alleviates the adverse effects of dyslipidemia on kidney function. Additionally, administration of 10 mg/kg bw/day of FIS in mice for 30 days supports healthy carbohydrate metabolism by enhancing the activity of critical enzymes, including hexokinase, pyruvate kinase, and glucose-6-phosphate dehydrogenase (G6PD). This increase in enzyme activity promotes efficient glucose processing and energy production and is essential for maintaining cellular health within the kidneys [45].

Furthermore, FIS reduces the enzymes associated with excessive gluconeogenesis, such as LDH, glucose-6-phosphatase, and fructose-1,6-bisphosphatase, while lowering blood glucose and HbA1c levels. This effect is particularly beneficial for individuals with diabetic nephropathy, as it improves blood sugar control and lessens metabolic strain on the kidneys [45, 51].

Beyond its metabolic improvements, FIS enhances levels of essential proteins and coagulation factors, including alpha-1 fetoprotein, alpha-2 macroglobulin, beta-2 microglobulin, transferrin, Alb, globulin, fibrinogen, and prothrombin. Moreover, Ge *et al.* revealed that FIS improves insulin sensitivity and reduces glucose intolerance, both critical for mitigating hyperglycemia-related stress on the kidneys. It also plays a critical role in reinforcing the glomerular filtration barrier by increasing levels of nephrin and podocin. These essential proteins maintain kidney structure and filtration function, which is often compromised in kidney disease [48]. Cellular protection prevents DNA damage within kidney cells, thereby preserving their function and longevity. This compound also reduces cellular degeneration and structural abnormalities, such as vacuolar degeneration and dilatation, which can adversely affect kidney cell health. By inhibiting receptor-interacting protein 3 (RIP3), a protein associated with necroptosis and inflammation, FIS further minimizes cellular death and inflammation, common contributors to kidney injury [48]. Additionally, Dong *et al.* indicated that FIS in 20 mg/kg of FIS for 8 weeks in diabetic mice enhances autophagy, the cell's natural cleanup process, by promoting CDKN1B/P70S6K-mediated pathways, thus safeguarding kidney cells from kidney injury [54].

Ren *et al.*, in their study, showed that 100 mg/kg of FIS for 4 weeks in mice with hyperuricemic nephropathy promotes renal uric acid excretion by modulating the expression of transporters such as ABCG2, OAT1, OAT3, and URAT1. These help maintain a balance of uric acid levels in the body and alleviate the strain associated with elevated uric acid on the kidneys. Notably, FIS reduces markers of podocyte injury while enhancing proteins vital for podocyte structural integrity, thereby aiding in preventing kidney disease progression [53].

In general, the mechanisms of FIS in reducing nephropathy caused by various pathogenic diseases can be seen in Fig. (**4**).

### 3.6 FIS Complications

Like many other flavonoids, FIS is generally considered well-tolerated and supposed to be a safe phytochemical, with minimal toxicity and few reported side effects [115, 116]. However, Krishnakumar *et al.*, in their randomized double-blinded study, reported that FIS supplementation was associated with minimal adverse events or clinical manifestations. Nonetheless, two instances of gastrointestinal discomfort were reported. Specifically, one subject experienced a sensation of bloating accompanied by a reduced appetite [117].

FIS can inhibit essential cytochrome P450 (CYP450) enzymes, particularly CYP3A4, CYP2C9, and CYP2C19, which are essential for metabolizing many common medications. This inhibition can lead to drug accumulation in the body, thereby increasing the risk of complications or toxicity, especially for drugs with narrow therapeutic ranges, including warfarin and certain statins [118]. FIS may exhibit prooxidant activity, a key mechanism behind their cytotoxic effects. Additionally, metabolic processes such as hydroxylation and oxidative demethylation, driven by cytochrome P-450 and O-methylation facilitated by catechol-O-methyltransferase, can substantially affect the cytotoxicity of the original compounds [119]. While FIS may induce some CYP450 enzymes under certain conditions, this interaction complicates drug metabolism [120]. Therefore, careful monitoring or dosage adjustments are necessary when taking FIS alongside medications that rely on CYP metabolism.

A recent preclinical study revealed that the FIS ruthenium-p-cymene complex had acute oral toxicity with a lethal dose of 50 (LD50) at 500 mg/kg. Subsequently, sub-acute doses were given to the subjects. The results from the sub-acute toxicity assessment showed that the group treated with 400 mg/kg had increased levels of white blood cells (WBC), Cr, aspartate aminotransferase, alkaline phosphatase, alanine aminotransferase, glucose, and cholesterol in their hematological and serum biochemical analyses [121].

## 4 DISCUSSION

This systematic review aimed to investigate the mechanistic effects of FIS on nephrotoxicity, kidney injury, and fibrosis induced by drugs, toxic chemicals, disorders, diet, ureteral obstruction, and an ischemic situation.

Nephrotoxicity refers to kidney injury or dysfunction caused by various factors, including chemical toxins, some medications, metabolic and immune diseases, or environmental exposures. Understanding the causes of nephrotoxicity and kidney injury is essential for effective prevention and management [110, 122]. Our study showed that during chemicals and drugs induced nephrotoxicity, IRI, and affecting systemic diseases, FIS enhanced the antioxidant defense system by eliminating ROS, improving the endogenous antioxidant defense system, and inhibiting pro-apoptotic and inflammatory pathways. Increased GFR, tubular regeneration, and reduced BUN, Cr, and fibrosis markers evidenced improvements in kidney function. Moreover, FIS reduces kidney tissue damage, improves histopathological changes, and contributes to nephroprotection. In line with our study, a study by Alsawaf *et al.* has indicated that flavonoids have protective effects on the kidneys by boosting antioxidant levels, reducing excessive ROS and DNA damage, and minimizing oxidative stress, mainly by acting as mediators in the Nrf_2_ antioxidant response.

Furthermore, flavonoids play a vital role in reducing drug and chemical toxicity. Flavonoids also alleviate inflammatory cytokines and prevent apoptosis, all of which contribute to preventing or improving kidney injuries and diseases [123]. Two other reviews also showed that FIS can alleviate cisplatin-induced nephrotoxicity by reducing oxidative stress and inflammatory cytokines levels, reducing Ca^2+^ influx, protecting against mitochondrial dysfunction, improving biological biomarkers, and reducing phosphorylation [22, 23].

Also, in our study, FIS had promising effects on reducing the complications of renal IRI. In line with these results, the study by Adeli *et al.* showed that FIS, through its antioxidant, anti-inflammatory, autophagy enhancement, mitochondrial protective effects, and anti-apoptotic characteristics, can alleviate renal cell damage caused by renal IRI [124]. So, through complex pathways and multifaceted effects, FIS significantly reduces renal dysfunction, injury, and fibrosis.

## 5 LIMITATIONS

While preclinical studies on FIS reported promising nephroprotective effects, there are limited clinical trials to support FIS as a treatment or complementary medicine for human kidney injury and nephropathy. The need for well-designed clinical trials is another limitation of the study. Ongoing trials, such as those listed on ClinicalTrials.gov, unpublished studies and non-English language articles, were not included in this systematic review. Despite the small number of these studies, missing these data could potentially impact the overall comprehensiveness and validity of our findings. Clinical trials could be useful for the translation of FIS into therapeutic applications for nephrotoxicity and renal injury. Moreover, the included studies did not investigate cytotoxicity and used different methods, chemicals, and drugs to induce nephrotoxic conditions and nephropathy.

## CONCLUSION

According to preclinical studies, FIS demonstrates promising protective effects against kidney toxicity, renal injury, and diabetes, as well as lupus-inducing nephropathy through multiple complex mechanisms that improve renal health and function. FIS exhibits significant nephroprotective effects by modulating oxidative stress, inflammation, apoptosis, fibrosis, and ferroptosis. Its antioxidant properties include the reduction of oxidative DNA damage markers, such as 8-OHdG, an increase in antioxidant enzymes (SOD, CAT, GR, and Nrf_2_/HO-1), and an enhancement of mitochondrial function and ATP production. Moreover, FIS's anti-inflammatory effects are characterized by the suppression of NF-κB, MAPK, and TLR4 pathways, leading to a decrease in pro-inflammatory cytokines (TNF-α, IL-6, IL-1β) and the inhibition of macrophage and neutrophil infiltration. Furthermore, it mitigates apoptosis by inhibiting Bax, caspases, and cytochrome c while promoting the expression of Bcl-2. The anti-fibrotic effects of fisetin include reduced deposition of ECM proteins, inhibition of TGF-β1/Smad signaling, and decreased COL1 expression, thus preserving kidney architecture. Fisetin enhances renal function by restoring GFR, lowering nephrotoxicity biomarkers (KIM-1, NGAL), and modulating biochemical markers such as BUN, Cr, and Alb. Additionally, FIS alleviates ferroptosis through the enhancement of GPX4 and regulates uric acid excretion *via* renal transporters. It also influences gut microbiota-derived tryptophan metabolites, including L-kynurenine, which is associated with decreased aryl hydrocarbon receptor expression in conditions of hyperuricemia.

It is recommended that future clinical studies examine different doses of this flavonoid and long-term implications, and examine possible side effects so that the results can be applied in the clinical field.

## Figures and Tables

**Fig. (1):**
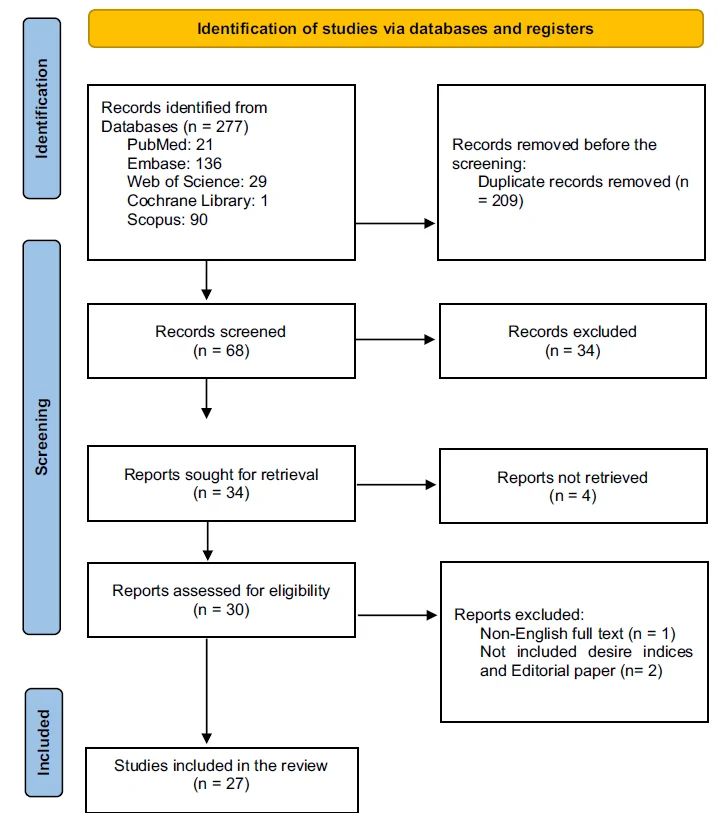
The diagram for screening studies into the systematic review.

**Fig. (2):**
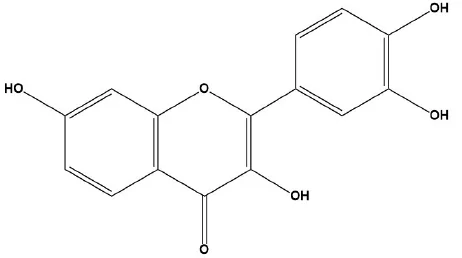
Chemical composition of FIS [38].

**Fig. (3):**
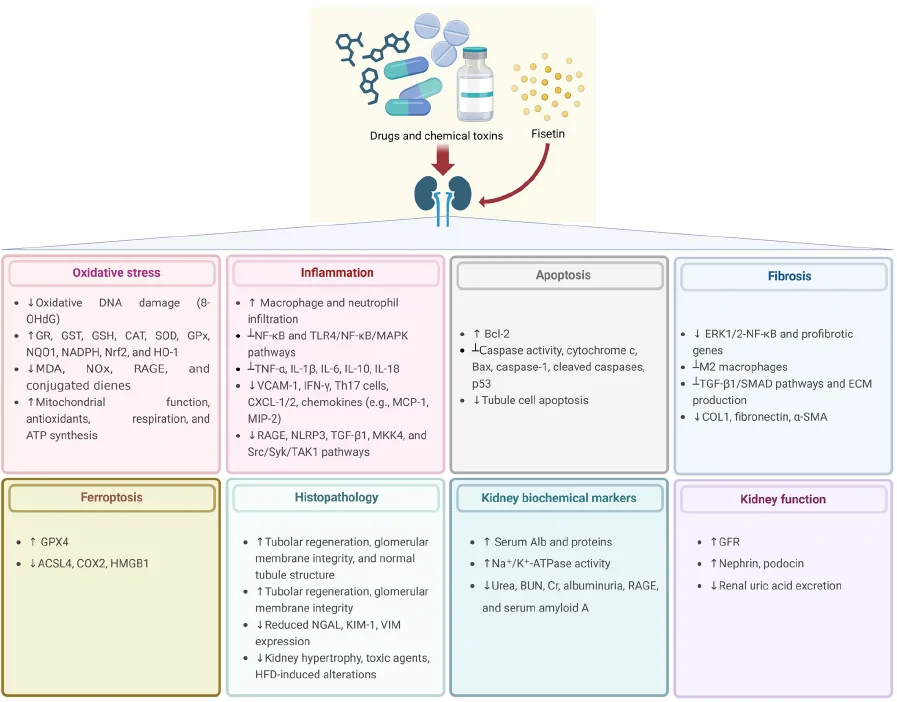
Fis's mechanisms against drugs and chemicals-induced nephrotoxicity. ┴: Inhibit; ↑: Increase; ↓: Reduce.

**Fig. (4):**
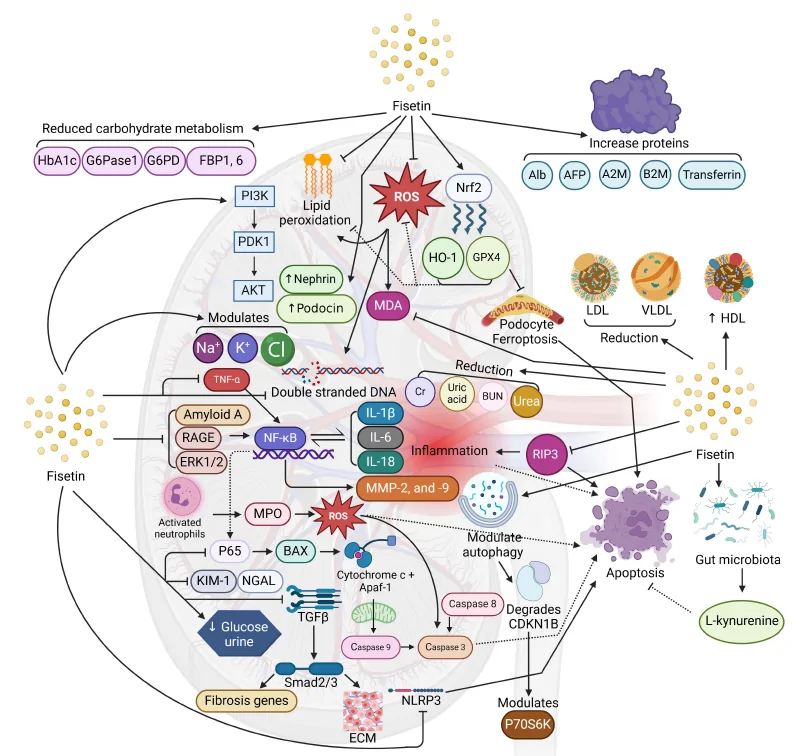
FIS demonstrates multiple nephroprotective pathways by alleviating oxidative stress (increasing GSH, GPx, CAT, SOD, Nrf_2_ and reducing MDA, and conjugated dienes), inflammation (reducing IL-1β, IL-6, TNF-α, NF-κB, NLRP3, and TAK1/p38MAPK/MK2), fibrosis (*via* TGF-β/SMAD and VEGFA pathways), and apoptosis (inhibiting caspases and BAX). It also reduces kidney hypertrophy, albuminuria, glycated proteins (*via* RAGE inhibition), serum amyloid A, and CRP. FIS improves mitochondrial function and Na^+^/K^+^-ATPase levels and restores nephrin and podocin integrity. Additionally, FIS modulates glucose metabolism (increased hexokinase, reduced glucose-6-phosphatase) and lipid profiles (decreased LDL, VLDL, increased HDL), reducing insulin resistance and glucose intolerance. It regulates uric acid excretion *via* ABCG2, OAT1, and URAT1, modulates gut microbiota to produce nephroprotective metabolites, and preserves renal structure, reducing markers of injury such as KIM-1, NGAL, and fibronectin.

**Table 1:** Characteristics of studies that investigated the nephroprotective effects of FIS against drugs and chemical-induced nephrotoxicity.

**Type of the Study**	**Chemical-Induced Nephrotoxicity**	**Studied Population /Animals/Cells**	**Treatment Details (Dosage, Duration)**	**Main Therapeutic Mechanisms**	**Lead Author/ Reference**
*In vivo*	CP	Male Sprague-Dawley rats	1.25 and 2.5 mg/kg of FIS was applied intraperitoneally for 7 consecutive days.	↑ SOD, GR, GST, CAT, NQO1, Bcl-2, mitochondrial antioxidants capacity, NADH dehydrogenases, SDH, and cytochrome c oxidase ↓ NADPH oxidase, cytochrome c protein, Bax, cleaved caspase-3, cleaved caspase-9 and p53 ┴ NF-κB, iNOS, and MPO	Sahu [35]
*In vivo* and *in vitro*	CP	C57BL/6 mice, BUMPT cells	100 mg/kg of FIS for 4 or 8 weeks.	↓ SCr, tubular expression of VIM, COL1, fibronectin, and ECM proteins ↑ GFR and restoration of tubular regeneration	Li [39]
*In vitro*	LPS	Rat mesangial HBZY-1 cells	0.05-200 μM of 6-Hydroxyflavone for 48 h.	↓ Mesangial cells, and iNOS	Wang [36]
*In vivo*	LPS	Male C57BL/6 J mice	100 mg/kg of FIS orally applied for 3 consecutive days.	↓ NGAL and KIM-1, iNOS, IL-6, IL-1β, TNF-α, HMGB1, and COX-2, TLR4, NF-κB, MAPK (p38, ERK1/2 and JNK), Bcl-2, Bax, and caspase-3	Ren [37]
*In vivo* and *in vitro*	LPS	BALB/c mice	10, 30, and 100 μM of FIS for 3 h.	↓ MCP-1, IL-1β, iNOS, MAPK (p38, ERK1/2 and JNK) ↓ uPA, uPAR, MMP-2, and MMP-9	Hada [38]
*In vivo*, *in vitro* and *ex vivo*	LPS	RAW264 cells	0-20 μM of FIS for 30 min.	↓ COX-2, and PGE2 ┴ ATP-competitive MKK4, Src, Syk, and TAK1-mediated pathways	He [41]
*In vivo* and *in vitro*	Lead Acetate	Male Wistar albino rats	100-200 mg/kg of FIS for 28 days.	↓ MDA and NOx levels, TNF-α, IL-6, IL-1β, BUN, Cr, and kidney tissue lead level ↑ SOD and CAT, GSH-Px, GSH, Nrf_2_, HO-1, and Zinc levels	Berköz [40]

**Note:** ┴, Inhibit; ↑, Increase; ↓, Reduce; **Abbreviations: ** LPS, Lipopolysaccharide; CP, Cisplatin; FIS, Fisetin; GR, Glutathione reductase; GST, Glutathione S-Transferase; CAT, Catalase; SOD, Superoxide Dismutase; NQO1, Nicotinamide Adenine Dinucleotide Phosphate (NADPH) Quinone Oxidoreductase 1; NF-κB, Nuclear Factor kappa-light-chain-enhancer of activated B cells; iNOS, Inducible Nitric Oxide Synthase; MPO, Myeloperoxidase; BCL-2, B cell lymphoma-2; SDH, Succinate dehydrogenase; NGAL, Neutrophil Gelatinase-Associated Lipocalin; KIM-1, Kidney Injury Molecule-1; MMP, Matrix Metalloproteinase-2; HMGB1, High Mobility Group Box 1; TNF-α, Tumor necrosis factor alpha; IL, Interleukin; COX-2, Cyclooxygenase-2; MAPKs, Mitogen-Activated Protein Kinases; TLR4, Toll-Like Receptor 4; ERK, Extracellular Signal-Regulated Kinases; JNK, c-Jun N-terminal Kinase; VIM, Vimentin; COL1, Collagen type I; ECM, Extracellular matrix; Cr, Creatinine; BUN; Blood urea nitrogen; MDA, Malondialdehyde; NOx, Nitric Oxide Metabolites; GSH-Px, Glutathione peroxidase; Nrf_2_, Nuclear factor erythroid 2-related factor 2, TAK1; Transforming growth factor-beta-activated kinase 1.

**Table 2:** Characteristics of studies that investigated the nephroprotective effects of FIS against renal IRI.

**Type of the Study**	**Renal IRI /Obstructive Nephropathy**	**Studied Population /Animals/Cells**	**Treatment Details (Dosage, Duration)**	**Main Therapeutic Mechanisms**	**Lead Author/ Reference**
*In vitro*	Renal IRI	LLC-PK1 cells	Cells were treated with bioflavonoids (50-100 µmol/L FIS) and cold stored for 20 hours.	↓ Cell injury and revealed renoprotective and cytoprotective properties against IRI	Ahlenstiel [42]
*In vitro*	Renal IRI	LLC-PK1 cells	Cells were treated with bioflavonoids (50-100 µmol/L FIS) and cold stored for 20 hours.	↓ Cell injury and cold ischemia	Ahlenstiel [43]
*In vivo* and *in vitro*	Renal IRI	Male Wistar rats	Administered 20 mg/kg b.wt, ip FIS for 30 minutes before the surgery.	↓ Renal damage due to IRI, oxidative stress in the kidneys, plasma Cr level, urinary Alb/Cr ratio, caspase activity, DNA fragmentation, and tubular damage score ↑ Antioxidant capabilities, mitochondrial function, and ATP synthase function	Prem [56]

**Note:** ┴, Inhibit; ↑, Increase; ↓, Reduce; **Abbreviations: **IRI, Ischemia/reperfusion injury; LLC-PK1, Epithelial-like pig kidney cell line; FIS, Fisetin; UUO, Unilateral ureteral obstruction; Cr, Creatinine; Alb, Albumin; 8-OHdG, 8-Hydroxy-2'-deoxyguanosine; GSH, Glutathione; GSSG, Glutathione disulfide.

**Table 3:** Characteristics of studies that investigated the nephroprotective effects of FIS against disorders-induced nephropathy.

**Type of the Study**	**Disorders-induced Nephropathy or Renal Injury**	**Studied Population /Animals/Cells**	**Treatment Details (Dosage, Duration)**	**Main Therapeutic Mechanisms**	**Lead Author/ Reference**
*In vivo* and *in vitro*	DN	C57BL/6-Ins2 (Akita) and SV40 MES13 cells	25-40 mg/kg of FIS for 6 weeks.	↓ Kidney hypertrophy, albuminuria, glycated by methylglyoxal, RAGE, and serum amyloid A, CRP ↑ GSH (by glyoxalase 1 enzyme)	Maher [44]
*In vivo*	DN	Male albino Wistar rats	10 mg/kg bw/day of FIS for 30 days.	↑ Hexokinase, pyruvate kinase, G6PD ↓ LDH, glucose-6-phosphatase and fructose-1, 6-bisphosphatase	Prasath [45]
*In vivo*	DN	Male albino Wistar rats	10 mg/kg bw/day of FIS for 30 days.	↓ LDL, VLDL ↑ HDL	Prasath [46]
*In vivo* and *in vitro*	DN	C57BL/6 mice and HK2 cells	20, 40, or 80 mg kg^−1^ of FIS for 16 weeks.	↓ Insulin resistance and glucose intolerance, HFD-induced histological alterations, KIM-1, Phosphorylated IRS1, TNF-α, IL-6, IL-1β, IL-18, RIP3, NLRP3, ASC, Caspase-1 ┴ NF-kB ↑ Nephrin and podocin, phosphorylated glycogen synthase kinase 1, AKT, FOXO1, GSK-3β	Ge [48]
*In vivo*	DN	Male Sprague Dawley rats	2.5, 5, and 10 mg/kg of FIS for 6 weeks.	↑ Body weight, serum Alb, and its excretion, ↓ Blood glucose level, lipid profile, serum urea, Cr, HbA1c, urine volume, urine Alb, TNF-α, IL-6, IL-1β, and histopathological changes	Kumari [51]
*In vivo* and *in vitro*	DN	Male eNOS homozygous knockout (eNOS^−/−^) mice and immortalized mouse podocytes	5, 10, or 20 mg/kg of FIS for 8 weeks.	↑ Autophagy-mediated CDKN1B/P70S6K ┴ NLRP3	Dong [54]
*In vivo* and *in vitro*	DN	eNOS homozygous knockout (eNOS^-/-^) mice and podocytes	5, 10, 20 mg/kg bw/day of FIS for 8 weeks.	↓ Glomerular injury ┴ Podocyte injury marker proteins ↑ Podocyte functional integrity marker proteins, Nrf_2_/ HO-1/GPX4	Qian [59]
*In vivo* and *in vitro*	DN	LDLR^−/−^ mice and HK-2 cells	30 mg/kg b.wt of FIS for 6 and 12 weeks.	↓ Atherosclerosis-enhanced diabetic kidney injury, urine glucose levels, ERK1/2-NF-κB pathway, IL-1β, IL-6 and TNF-α, VCAM-1, serum urea, uric acid, and Cr ┴ CD36 expression and at the following inhibited ECM accumulation (by inhibiting VEGFA and fibronectin and collagens)	Zou [60]
*In vivo*	Hyperuricemic nephropathy	C57BL/6J mice	50 and 100 mg/kg of FIS for 4 weeks.	↓ Uric acid, serum Cr, and BUN, NGAL, KIM-1, fibronectin, Col1a1, and α-SMAModulate the structure and composition of gut microbiota ↑ Nephroprotective by tryptophan metabolites produced by gut microbiota	Ren [52]
*In vivo*	Hyperuricemic nephropathy	C57BL/6J mice and TCMK-1 cells	50 and 100 mg/kg of FIS for 4 weeks.	↓ Hyperuricemia, urinary Alb/Cr ratio, IL-1β, IL-6 and TNF-α, MCP-1, uric acid-stimulation ↑ Tissue architecture, Regulated ABCG2, OAT1, OAT3 and URAT1, expressions ┴ TGF-β/SMAD3	Ren [53]
*In vivo* and *in vitro*	Lupus	C57BL/6 mice RAW264.7 murine macrophage cell line and the 293FT cell line	50 and 100 mg/kg of FIS for 30 weeks in mice 0-160 µM of FIS for 24-72 h in cells.	↓ Anti-small nuclear ribonucleoprotein, Pristane-induced anti-double-stranded DNA, Alb, Cr, CXCL-1 and 2, chemokine (C-C motif) ligand 3, as well as CXC receptor 2, IL-6, IFN-γ, TNF-α, IL-1β, and McP-1 ┴ Th17 cells	Xu [47]
*In vivo* and *in vitro*	Lupus	Female MRL/lpr mice and TECs	100 mg/kg of FIS in mice 5, 10, or 20 µM of FIS for 2 days in cells.	┴ TGF-β and following senescence of NRK-52E renal TECs and proliferation of NRK-49F renal fibroblasts	Ijima [55]
*In vivo* and *in vitro*	Obstructive nephropathy	C57BL6 mice and HK-2 cells	Administered 25 mg/kg; Pytolab of FIS 1 hour before operation and on days 2, 4, and 6 after it.	┴ Profibrotic M2 macrophages, phosphorylation of SMAD3 and SMAD2 induced by TGF-β1 ↓ 8-OHdG, macrophage infiltration, neutrophils, MCP-1, MIP-2, and IL-1β, tubule cell apoptosis	Ju [57]
*In vivo* and *in vitro*	Diet and UUO	CKD models in adult male mice and TCMK-1 cells	50 or 100 mg·kg (-1) ·d (-1), i.g. of FIS in mice for 3 weeks 0-40 μM for cells for 7 days.	┴ Tubular ferroptosis (reducing ACSL4), adenine- or TGF-β1-induced inflammatory responses and fibrogenesis. ↑ Mitochondrial morphology, GSH and GSH/GSSG ↓ MDA (Lipid peroxidation)	Wang [61]
*In vivo* and *in vitro*	High-fat diet-induced nephropathy	Male C57BL/6 mice	20, 40, and 60 mg/kg of FIS for 16 weeks in mice 10, 20 and 40 μM of FIS in 24 h for cells.	↑ Nrf-2/HO-1 ┴ iRhom2/NF-κB signaling pathway	Chenxu [50]
*In vivo* and *in vitro*	Sepsis	Male C57BL/6 mice and HEK293T cells	10 mg/kg of FIS for 18 in mice 0, 3, 10, or 30 μM of FIS for 6 h in cells.	┴ IL-1β, IL-6, TNFα, TAK1/p38MAPK/MK2 signaling pathways	Zhang [49]
*In vivo*	Diarrhea	Male albino Sprague-Dawley	50 mg/kg of FIS for 4 weeks.	↓ Serum urea, Cr, urinary volume, MDA, conjugated dienes, oxidative index, IL-1β, IL-6, IL-10, TNFα, and kidney DNA damage ↑ Kidney weight, GPx, CAT, SOD, NADPH, Na^+^/K^+^-ATPase levels and protein contents Modulated sodium, potassium, and chloride	Koriem [58]

**Note:** ┴, Inhibit; ↑, Increase; ↓, Reduce; **Abbreviations: ** DN, Diabetic neuropathy; FIS, Fisetin; LLC-PK1; Pig Kidney Epithelial Cells, IRI; Ischemia-reperfusion injury, ATP; Adenosine three phosphate; RAGE, Receptor for advanced glycation end products; CRP, C-reactive protein; HDL, high-density lipoprotein; LDL, Low-density lipoprotein; VLDL, Very LDL; LDH, Lactate dehydrogenase; G6PD, Glucose-6-phosphate dehydrogenase; HFD, High fat diet; KIM-1, Kidney Injury Molecule-1; AKT, FOXO1; Forkhead box protein O1; GSK-3β, Glycogen Synthase Kinase-3 Beta; RIP3, Receptor Interacting Protein 3; NLRP3, NOD-like receptor protein 3; Alb, Albumin; Cr, Creatinine; VCAM-1, Vascular Cell Adhesion Molecule-1; α-SMA, Alpha-smooth muscle actin; MCP-1, Monocyte chemoattractant protein-1; IFN-γ, Interferon-γ; GPx, Glutathione peroxidase; NADPH, Nicotinamide adenine dinucleotide phosphate hydrogen; MDA, Malondialdehyde; VEGFA, Vascular endothelial growth factor A.

**Table 4:** Some critical drugs derived from natural resources.

**Drug Name**	**Therapeutic Application**	**Natural Origin**	**Reference**
Aspirin	Analgesic, CVDs, Anti-inflammatory	Willow tree and salicin (salicylic acid)	[65]
Morphine	Analgesic	Alkaloid is derived from opium and is the prototype opiate	[23]
Paclitaxel (Taxol)	Neoplasm treatment	Pacific yew tree, *Taxus brevifolia*	[66]
Vincristine	Neoplasm treatment	Alkaloid is derived from *Catharanthus roseus*	[67]
Quinine	Treatment of malaria	Alkaloid from *Cinchona officinalis*	[68]
Penicillin	Antibiotic	Secondary metabolite from some fungi, including *Penicillium chrysogenum* and *Penicillium nalgiovense*	[69]
Digoxin	Heart failure, arrhythmias	Cardiac glycoside derived from *Digitalis lanata*	[70]
Atropine	Eye dilation, bradycardia	Alkaloid is derived from *Atropa belladonna*	[71]

**Abbreviation: ** CVD, Cardiovascular disease.

## Data Availability

The authors confirm that the data supporting the findings of this research are available within the article.

## LIST OF ABBREVIATIONS

8-OHdG: 8-Hydroxy-2'-Deoxyguanosine
AKT: Protein Kinase B
Alb: Albumin
α-SMA: Alpha-Smooth Muscle Actin
ATP: Adenosine Triphosphate
BCL-2: B-Cell Lymphoma-2
BUN: Blood Urea Nitrogen
CAT: Catalase
COX-2: Cyclooxygenase-2
CP: Cisplatin
Cr: Creatinine
CRP: C-Reactive Protein
DN: Diabetic Nephropathy
ECM: Extracellular Matrix
ERK: Extracellular Signal-regulated Kinase
FIS: Fisetin
FOXO1: Forkhead Box Protein O1
G6PD: Glucose-6-Phosphate Dehydrogenase
GPx/GSH-Px: Glutathione Peroxidase
GR: Glutathione Reductase
GSH: Glutathione
GSSG: Glutathione Disulfide
GSK-3β: Glycogen Synthase Kinase-3 Beta
GST: Glutathione S-Transferase
HDL: High-density Lipoprotein
HFD: High-fat Diet
HMGB1: High Mobility Group Box 1
HO-1: Heme Oxygenase-1
IFN-γ: Interferon-gamma
IL: Interleukin
iNOS: Inducible Nitric Oxide Synthase
IRI: Ischemia/Reperfusion Injury
JNK: c-Jun N-terminal Kinase
KIM-1: Kidney Injury Molecule-1
LDH: Lactate Dehydrogenase
LDL: Low-Density Lipoprotein
LLC-PK1: Epithelial-like Pig Kidney Cell Line
LPS: Lipopolysaccharide
MAPKs: Mitogen-activated Protein Kinases
MCP-1: Monocyte Chemoattractant Protein-1
MDA: Malondialdehyde
MMP: Matrix Metalloproteinase-2
NADPH: Reduced Nicotinamide Adenine Dinucleotide Phosphate
NF-κB: Nuclear Factor Kappa-Light-Chain-Enhancer of Activated B Cells
NGAL: Neutrophil Gelatinase-Associated Lipocalin
NOx: Nitric Oxide Metabolites
NLRP3: NOD-Like Receptor Protein 3
NQO1: NAD(P)H Quinone Dehydrogenase 1
Nrf_2_: Nuclear Factor Erythroid 2-Related Factor 2
RAGE: Receptor for Advanced Glycation End Products
RIP3: Receptor-Interacting Protein 3
SDH: Succinate Dehydrogenase
SOD: Superoxide Dismutase
TAK1: Transforming Growth Factor-Beta-Activated Kinase 1
TLR4: Toll-Like Receptor 4
TNF-α: Tumor Necrosis Factor-Alpha
UC/UUO: Unilateral Ureteral Obstruction
VCAM-1: Vascular Cell Adhesion Molecule-1
VEGFA: Vascular Endothelial Growth Factor A
VIM: Vimentin
VLDL: Very Low-density Lipoprotein

## STANDARDS OF REPORTING

PRISMA guidelines were followed.

## SUPPLEMENTARY MATERIAL

PRISMA checklist is available as supplementary material on the publisher’s website along with the published article.
